# Neonatal Skull Depression: The Role of Cranial Ultrasound

**DOI:** 10.7759/cureus.52872

**Published:** 2024-01-24

**Authors:** André Assunção, Filipa Flor-de-Lima, Josué Pereira, Daniela Pinto

**Affiliations:** 1 Department of Pediatrics, Centro Hospitalar Universitário São João, Porto, PRT; 2 Department of Neonatology, Centro Hospitalar Universitário São João, Porto, PRT; 3 Faculty of Medicine, University of Porto, Porto, PRT; 4 Department of Pediatric Neurosurgery, Neuroscience Center, CUF Hospital, Porto, PRT; 5 Department of Pediatric Radiology, Lusíadas Hospital, Porto, PRT

**Keywords:** diagnostic modalities, skull defects, newborn, neonatal skull depression, cranial ultrasound

## Abstract

Nontraumatic congenital neonatal skull depression is a rare condition resulting from external forces shaping the fetal skull. Typically, newborns are asymptomatic, and, usually, the condition resolves in a few months with no need for intervention. However, many newborns undergo a CT scan, an ionizing technique, to check for fractures or intracranial lesions. We report a case of congenital skull depression without neurological deficits, managed conservatively through clinical monitoring and ultrasound.

## Introduction

Congenital skull depression (CSD) is a rare condition, with an incidence of 1-2.5 per 10,000 live births, either traumatic or nontraumatic, but its true cause is often unknown [1]. Most authors describe it as nontraumatic, as there is no traumatic event that it can be traced back to [2]. However, in a few cases, it will be related to a traumatic incident during pregnancy or delivery, and differentiating them is important for proper management. Nontraumatic CSD stems from external forces that shape the cartilaginous neonatal skull either during pregnancy (e.g., uterine fibroids, maternal pelvic or spinal bone prominences, or the body part of a twin) or during labor (e.g., instrumental delivery or because of the hands of the obstetrician) [1,2]. This condition induces parental concerns about potential cerebral damage, later neurodevelopmental issues, as well as cosmetic concerns [3]. Therefore, such a condition can be considered a family stressor. Computed tomography (CT) scan is considered the gold standard for the diagnosis of skull fractures and traumatic brain injury. However, it exposes infants to ionizing radiation, increasing the risk of cancer over a lifetime [4]. On the other hand, cranial ultrasound presents as a noninvasive and readily available imaging technique, promising for the assessment of skull anomalies, compression, atrophy or scarring of brain structures, and intracranial hemorrhages among other changes [5-8].

We present a case of a neonatal CSD, identified at birth, who was followed up clinically and with ultrasound scans, thus avoiding the need for exposure to ionizing radiation imaging techniques of a neonate with normal neurological examination and no red flags during reassessments. This case highlights the value of ultrasound in the evaluation and follow-up of CSD in neonates with normal neurological examinations.

## Case presentation

**Figure 1:**
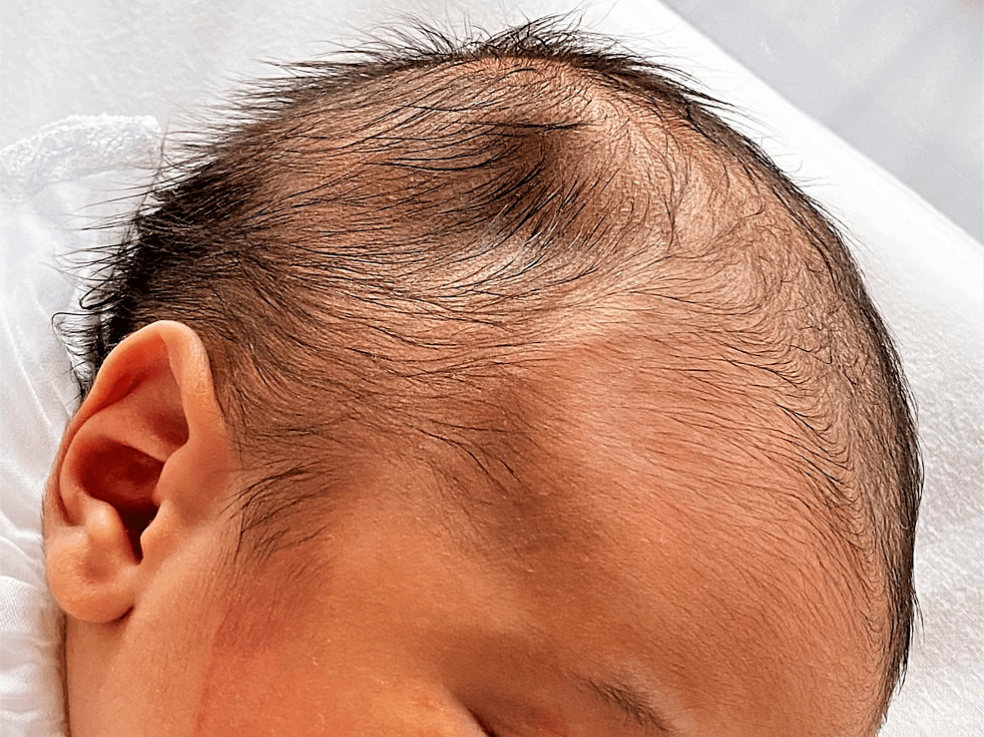
Neonatal congenital skull depression Skull depression had dimensions of 3 cm x 3 cm and a depth of less than 1 cm.

After an uneventful pregnancy, a female term newborn was delivered via elective cesarean, without instrumentation. There was no history of trauma during pregnancy or delivery. The newborn was vigorous and cried immediately after birth, and Apgar scores were nine and 10 at one and five minutes, respectively. At the first examination, a 3x3 cm right parietal skull depression (Figure 1) was noted, characterized as solid, nonpulsatile, and nonmobile. Otherwise, physical and neurological examinations were unremarkable. The newborn’s anthropometric measures were 2680g, 46cm in length, and 33 cm of head circumference. The infant’s overall status was good during the first hours of life, and she adapted well to breastfeeding. There was no change in behavior or neurological status, and no bruising or swelling of soft tissues suggestive of acute injury.

**Figure 2:**
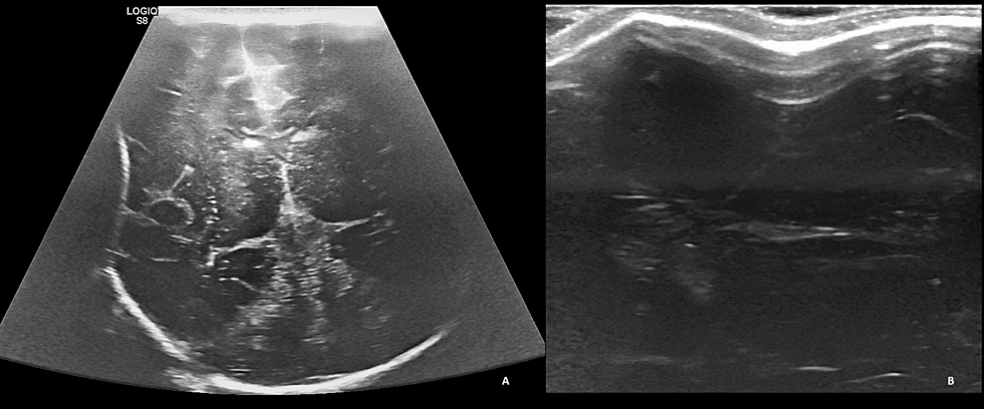
Cranial ultrasound of neonatal skull depression Performed on the third day of life and revealed a depression on the right parietal bone with no signs of fracture, intracranial structural anomalies, or bleeding (A-coronal view; B-M mode at the location of skull depression)

Given the absence of a history of trauma, signs of acute injury, and abnormal neurological findings, a consultation with a radiologist and a neurosurgeon led to the decision to perform cranial ultrasound for radiological assessment. The ultrasound, performed on day three of life by an experienced radiologist using high-frequency linear transducer probes (5-10 MHz), revealed an abnormal skull depression at the parietal region with an inward configuration, likely because of the molding of the parietal bone (Figure 2). Scans were performed in two perpendicular planes, recording pictures in both orientations, and the contralateral area on the skull was imaged for comparison. Skull fractures and suture lines were distinguished by following suspected sutures to a fontanelle, and there were no signs of fractures, of intracranial structural anomalies, or bleeding. As the most likely diagnosis was nontraumatic CSD in a newborn with normal neurological status, a conservative management approach was chosen, with frequent clinical and radiological follow-up.

At the first follow-up, at 15 days of age, the depression had already decreased in both depth and size. Both physical and neurological examinations remained unremarkable, and a second ultrasound at this time confirmed the favorable clinical findings, excluding any other intracranial abnormalities. Follow-up visits continued at one month, one and a half months, two months, and three months of age, during which the depression steadily decreased, and the infant exhibited no warning neurodevelopmental signs. By the fourth month, the previously diagnosed CSD had fully regressed both clinically and radiologically. The infant's development proceeded normally.

## Discussion

Nontraumatic CSD is a rare condition, and the main differential diagnosis is a fracture of the skull induced by obstetric procedures. The diagnosis of traumatic CSD should not be ruled out easily; however, in our case, given the delivery method was an elective and non-instrumentalized cesarean, an obstetric-related trauma does not seem likely. There are some reports of assisted births wherein the location of the CSD is different from ventouse or forceps applications [9,10], supporting the idea that an instrumentalized birth may not be a reason for such a CSD. Therefore, the most likely explanation is external pressure on the cartilaginous and moldable neonatal skull.

As in many reports, our newborn was born weighing over 2500g, although a CSD can also develop in smaller newborns [3]. The standard diagnostic imaging method is a CT scan; however, given the non-instrumentalized delivery, a normal physical and neurological examination, after consultation with a radiologist and a neurosurgeon, we chose to assess for cranial fractures and intracranial injuries using an alternative, easily available and nonionizing radiation tool, such as ultrasound.

Regarding management, most authors recommend a conservative approach as long as there is neither intracranial involvement nor physical or neurological deficits in the newborn [3,10,11]. Particularly in such instances, follow-up is essential to assess the evolution of the depression: whether it decreases with time or not and whether any physical or neurological signs develop that might lead to a change in management strategy. Moreover, imaging reassessment allows for documentation of changes in perfusion, with bleeding or cortical changes suggestive of local complications from CSD. Thus, a close follow-up should be mandatory in such cases for early identification of red flags. In our case, the newborn presented with no evidence of complication at birth; therefore, we opted for a conservative approach with regular follow-up. For initial imaging evaluation and follow-up, we chose ultrasound for its ready availability, noninvasiveness, and radiation-free nature that allows for the assessment of fractures and intracranial injuries with good predictive value [4,6,12].

During follow-up, we included the co-evaluation of a pediatric neurosurgeon to have both a first assessment and an early plan in case some intervention, surgical or not, would be needed. Usually, only traumatic and nontraumatic but large and deep depressions require corrections [1,4,13], but different options are available for different situations; therefore, the neurosurgical evaluation was requested. In our case, as surgical intervention was unnecessary, this second medical appointment ensured closer follow-up of the infant.

## Conclusions

Most cases of nontraumatic CSD, not associated with neurological impairment, intracranial bleeding, or neurodevelopmental abnormalities, will resolve spontaneously, and a conservative approach may be the appropriate choice. According to the literature, only severe cases may require neurosurgical intervention for correction.

This case emphasizes the viability of a conservative approach in the absence of neurological symptoms, using ultrasound for initial evaluation and close follow-up to identify early any signs that may suggest a deviation from the normal progression of this condition. Therefore, frequent medical appointments for clinical assessment and reevaluation are needed. A co-evaluation by a pediatric neurosurgeon is also recommended. More studies are recommended to evaluate the possibility of broadening the use of ultrasound for the assessment of such cases.
